# Deep Peroneal Nerve Palsy Caused by an Extraneural Ganglion Cyst: A Rare Case

**DOI:** 10.1155/2015/861697

**Published:** 2015-01-06

**Authors:** Dimitrios Nikolopoulos, George Safos, Neoptolemos Sergides, Petros Safos

**Affiliations:** ^1^Orthopaedic Department, Central Clinic of Athens, 31 Asklepiou Street, 10680 Athens, Greece; ^2^Orthopaedic Department, Ikaria General Hospital, 83302 Ikaria, Greece

## Abstract

Lower extremities peripheral neuropathies caused by ganglion cysts are rare. The most frequent location of occurrence is the common peroneal nerve and its branches, at the level of the fibular neck. We report the case of a 57-year-old patient admitted with foot drop, due to an extraneural ganglion of the upper tibiofibular syndesmosis, compressing the deep branch of the peroneal nerve. Although there have been many previous reports of intraneural ganglion involvement with the lower limb nerves, to our knowledge, this is the second reported occurrence of an extraneural ganglion distinctly localized to the upper tibiofibular syndesmosis and palsying deep peroneal nerve. The diagnosis was made preoperatively using MRI. The common peroneal nerve and its branches were recognized and traced to its bifurcation during the operation, and the ganglion cyst was removed. Two months after surgery, the patient was pain-free and asymptomatic except for cutaneous anesthesia in the distribution of the deep peroneal nerve.

## 1. Introduction

Ganglion cysts are the most frequent tumors of the upper and lower extremity. Despite their high incidence, ganglion cysts rarely result in peripheral nerve compression [1]. According to the English literature, previous reports of intraneural ganglion involvement with the common peroneal nerve and its branches, the sural nerve, and the posterior tibial nerve have been described, whereas extraneural ganglion sciatic and common peroneal nerve palsy cases are scarce [2–6]. However, leg pain, sensory loss, and weakness in ankle dorsiflexion due to deep peroneal nerve palsy from an extraneural ganglion distinctly localized to the upper tibiofibular syndesmosis have not been reported until nowadays. Historically, the first reported case of compression neuropathy to peroneal nerve by a synovial cyst was by Sultan in 1921 [7], whereas the first reported rare cause of deep peroneal nerve palsy due to compression of synovial cyst was by Erdil et al. in 2013 [8].

The common peroneal nerve is derived from the dorsal branches of the fourth and fifth lumbar and the first and second sacral nerves. It descends obliquely along the lateral side of the popliteal fossa to the head of the fibula, close to the medial margin of the biceps femoris muscle. The common peroneal nerve (CPN) winds round the head of the fibula and divides beneath the muscle into the superficial peroneal nerve (SPN) and the deep peroneal nerve (DPN). The SPN supplies the peroneus longus and peroneus brevis muscles. The DPN innervates the muscles of the anterior compartment of the lower extremity, such as tibialis anterior, extensor hallucis longus, extensor digitorum longus, and the fibularis (peroneus) tertius. Together these muscles are responsible for dorsiflexion of the foot and extension of the toes. It also innervates intrinsic muscles of the foot including the extensor digitorum brevis and the extensor hallucis brevis [5, 6, 9, 10].

We report the case of a 57-year-old patient admitted to our clinic with foot drop since 4 weeks. At first the deep peroneal nerve palsy was misdiagnosed as being L5-S1 disk herniation. After electromyography (EMG) of the lower extremities and knee magnetic resonance imaging (MRI), an extraneural ganglion of the upper tibiofibular syndesmosis was diagnosed, compressing the deep branch of the peroneal nerve.

## 2. Case Presentation

A 57-year-old Caucasian male presented to his physician with an acute onset of left lower limb pain and sudden foot drop. The patient referred to numbness and motor weakness in the lateral aspect of his left leg, after the onset of severe pain. His medical history revealed a single episode of low back pain without numbness, motor weakness, and severe pain four years before. The physician resulted in the misdiagnosis of degenerative disc disease of the lumbar spine and recommended MRI of the lumbar spine and prescribed painkillers and anti-inflammatory treatment for one week. Lumbar's spine MRI was normal, whereas one week after the onset of the symptoms, there was no clinical improvement.

The patient visited our clinic 4 weeks after the onset of the symptoms. He was unable to perform foot eversion and dorsiflexion, especially of the first toe, when he was referred to our hospital. Inversion was normal. Manual muscle testing of the left tibialis anterior, extensor hallucis longus, extensor digitorum longus, and peroneus muscles revealed muscle strength at the level of 2 (trace), according to the Daniels and Worthingham muscle power grading system [11]. Diminished sensation in the first web space and positive Tinel's sign near the head of the fibula on the left foot were also detected during the examination. Tenderness in the area of the left fibular head with gradual development of swelling in the same area was determined, although no mass lesion was palpable.

**Figure 1:**
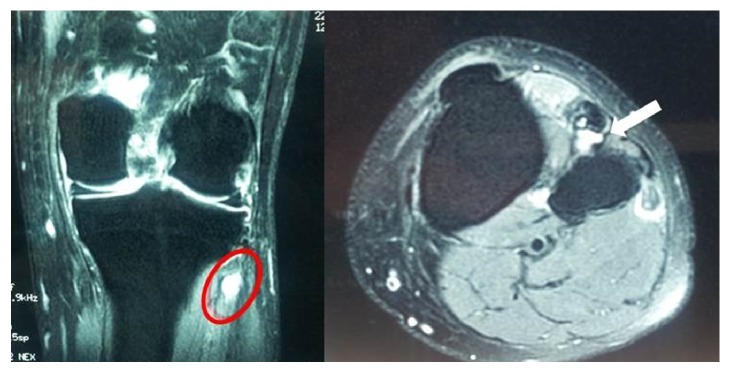
Left knee MRI demonstrated a lobulated, multilocular, and well-demarcated cystic-appearing mass medial to the fibular head (ganglion).

Electromyogram studies of the CPN and its branches demonstrated significant neuropathic abnormalities of the DPN. No abnormality was found in the muscles innervated by the tibial nerve. Subsequent left knee MRI demonstrated a lobulated, multilocular, and well-demarcated cystic-appearing mass medial to the fibular head. It measured approximately 3.2 cm × 2.5 cm × 2 cm (Figure 1). The lesion was located anteromedial to the fibular neck, anterior of the upper tibiofibular syndesmosis, compressing the DPN.

**Figure 2:**
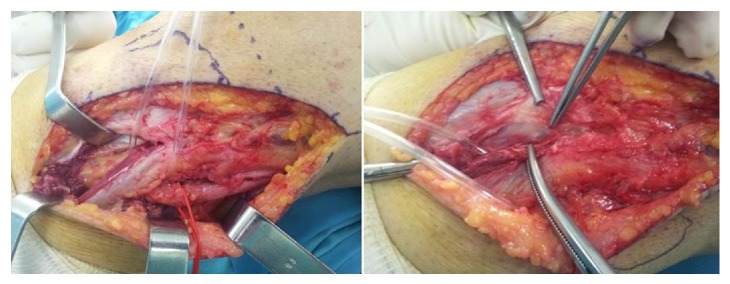
Left knee lateral approach. The CPN and its branches were recognized and traced to its bifurcation. The ganglion mass was followed down to its stalk and removed completely.

With a lateral approach, the CPN and its branches were recognized and traced to its bifurcation (Figure 2). The mass was followed down to its stalk and removed completely. The DPN was recognized as intact. All nerve branches were preserved under loupe magnification. The tumor had no connection with the proximal tibiofibular joint or with the knee joint. Histopathological evaluation of the decompressed gelatinous material verified the diagnosis of ganglion.

Postoperatively, he was treated with an antifoot drop polyethylene splint, fixed in neutral ankle position, and physiotherapy. Forty-eight hours postoperatively the patient had no complaints of pain, but the foot drop remained although the sensation in the first web space improved (4/5). The patient recovered to grade 4 toe extension and ankle dorsiflexion within six weeks after the surgery. Two months after operation there was an almost full recovery of the motor function with minimal numbness in the first web space.

## 3. Discussion

Peripheral nerve lesions owing to ganglionic cysts are infrequent findings [2]. Since the first description of a peroneal nerve neuropathy by Sultan in 1921 [7], a minority of cases of the lower extremity compression neuropathies have been described in the surgical literature [1–7, 10–12]. The CPN, derived from L4, L5, S1, and S2 as a main division of the sciatic nerve, arises near the upper level of the popliteal fossa and becomes most vulnerable at its entrance to the fibular tunnel, where it courses superficial to the lateral surface of the fibula just distal to the fibular head, having little soft tissue protection [1, 7]. The proximal tibiofibular joint consists of the articulating surfaces of the upper end of the fibular head and the tibial lateral condyle. Both these surfaces are covered with hyaline cartilage. This joint is lined by a synovial membrane and is often in communication with the knee joint. The joint is surrounded by an articular capsule which is reinforced by the anterior and posterior ligaments [13]. Rawal et al. [4] hypothesized that the origin of peroneal nerve ganglia is the proximal tibiofibular joint, via the articular branch.

A review of the literature supplies an extensive list of etiologies for peroneal nerve palsy, intraneural (schwannoma, nerve herniation through a fascial defect, and giant plexiform neurofibromatosis) [1, 12, 14–16] and extraneural (varicose veins and pneumatic compression, after knee surgical operation such as a valgus knee total arthroplasty or proximal tibial osteotomy and ganglion cysts) [1, 2, 4, 14, 17]. Although rare, intraneural ganglions located within the substance of nerves may cause direct nerve compression with acute onset of symptoms [5]. As already mentioned, there have been many previous reports of intraneural ganglion involvement with the CPN and its branches [3, 5, 12]; however, this is the second reported occurrence of an extraneural ganglion distinctly localized to the upper tibiofibular syndesmosis and palsying DPN.

Ganglion cysts are commonly accompanied by signs of nerve irritation such as numbness, tingling, and pain in the distribution of the affected nerve. In peroneal nerve palsies, the patients often complain of altered ambulation secondary to paretic or paralyzed ankle dorsiflexors. A steppage gait pattern is common, due to the weakness in the tibialis anterior, the extensor hallucis longus, the extensor digitorum longus, and the peroneus longus and brevis muscles. The affected foot requires extra lift from the ground during the swing phase of ambulation to clear the foot [1, 2]. The extensor digitorum brevis is the most profoundly affected. The tibialis anterior can also be significantly affected with weakness in ankle and toe dorsiflexion. Often, ankle eversion is normal, because patients can have relative sparing of these muscles [1, 2]. While pain is not universal, when present, it is often related to the specific site of the CPN compression or its branches (superficial or deep). Sensory testing often shows a loss in the cutaneous distribution of the superficial and deep peroneal nerves. Tinel's sign is generally positive in the sensory distribution of the peroneal nerve [1].

The differential diagnosis should include L5 root pathology (compression), a nerve compression near the tendinous arch of the peroneal longus muscle, a nerve-sheath tumor, the osteocartilaginous exostosis at the proximal lower leg [6, 10, 12, 18, 19], and intermittent claudication [2]. Plain radiographs have little importance in the diagnosis of ganglion cyst but may be beneficial in eliminating a bony anomaly or fracture of the proximal part of fibula. Furthermore, they may be useful in excluding degenerative disc disease of the lumbar spine [1, 10]. An EMG study may be helpful to distinguish the level of the palsy and the extent of sensory and motor impairment. MRI is the noninvasive technique of choice for diagnostic imaging. It may be difficult to differentiate a ganglion cyst from nerve sheath tumors and also solid masses on magnetic resonance imaging. Ganglia characteristically present with low signal on T1-weighted images and high signal on T2-weighted images [20]. Ultrasonography (US) may be effective in showing the cystic nature of the mass (well-circumscribed) and in differentiating it from solid tumors (anechoic lesion) [1, 10, 21–23]. Although US is a noninvasive and cheap screening method, it is not sensitive enough to distinguish ganglia from other nerve sheath tumors [8]. Nevertheless, Visser [22] and Bayrak et al. [23] presented that sonographic detection of multifocal nerve enlargement is useful for the diagnosis of hereditary neuropathy with liability to pressure palsies especially when electrodiagnostic studies are limited and/or genetic analysis is not compatible.

Currently, the gold standard treatment for a peroneal nerve palsy due to a peripheral nerve ganglion compression is surgical removal of the ganglion [1–5, 12–17]. Careful surgical planning and proper delineation of the mass is vital in identifying its origin. It is important to excise the stalk and its base in the superior tibiofibular joint so as to lessen the risk of recurrence. Sometimes the small sensory articular branch of the joint has to be sacrificed [4]. The prognosis of the peroneal nerve palsies of compressive origin is excellent. When conservatively treated, recovery may take 1 year or 2 years and it may be incomplete, requiring the patient to use a peroneal brace. Instead recovery is much faster after operative decompression and will take place after few days or a few weeks [3, 13, 14]. In our case the patient recovered to grade 4 toe extension and ankle dorsiflexion within six weeks after the surgery, and two months after operation there was an almost full recovery of the motor function with minimal numbness in the first web space.

Fabre et al. [24] reported 60 patients with peroneal nerve palsies, many idiopathic, who were treated with operative decompression. Postoperative recovery of motor function was good to excellent in 87% of those who had both sensory and motor involvement preoperatively. Decompression is recommended even for patients presenting with only sensory symptoms, if the symptoms have been substantiated by electrophysiological studies. Local cyst recurrence postoperatively is reported and stresses the importance of articular branch ligation to avoid this complication [1, 20, 25, 26]. Simple excision of the ganglia is not sufficient. Other complications include traction injuries and perineural fibrosis with incomplete return of function. Less commonly, nerve transection occurs, leading to permanent dysfunction [1].

## 4. Conclusions

Ganglion cysts should be considered in the differential diagnosis of progressive peroneal or sciatic nerve palsy. Other clinical entities that should be considered in the differential diagnosis include L5 root lesions and nerve sheath tumors. After a complete history and physical examination, EMG and MRI should be performed in terms of the differential diagnosis of a ganglion cyst. Careful preoperative evaluation and early surgical excision, including also decompression of the nerve by microsurgical technique in the management of the ganglion cyst, should be recommended.
